# Effect of a nursing intervention based on nutritional support on biochemical parameters and quality of life in patients with hyperthyroidism combined with diabetes mellitus

**DOI:** 10.3389/fendo.2025.1629902

**Published:** 2025-11-04

**Authors:** Huanjing Yang, Jiajia Feng

**Affiliations:** Endocrine Department, Nanjing First Hospital, Nanjing Medical University, Nanjing, Jiangsu, China

**Keywords:** biochemical parameters, diabetes mellitus, hyperthyroidism, nursing intervention, nutritional support, quality of life

## Abstract

This study was conducted to study the effect and of nursing intervention on biochemical parameters, complications and quality of life in patients with hyperthyroidism combined with diabetes mellitus. This study included 112 patients who were treated for diabetic hyperthyroidism from April 2022 to April 2024 in our hospital. The control group was given routine care, and the nursing group was given nutritional, psychological and exercise nursing interventions on the basis of the control group. The blood glucose, thyroid function, nutritional status, complications, nursing satisfaction and quality of life of patients in different groups were compared. The analysis show that nursing intervention can effectively improve the patients’ blood glucose level, thyroid function and nutritional status, and reduce the incidence of complications so as to improve the patients’ quality of life. In conclusion, nursing intervention based on nutritional support can successfully upgrade the condition and prognosis of patients with hyperthyroidism combined with diabetes mellitus and improve nutritional status and quality of life.

## 1 Introduction

Diabetes mellitus (DM) is one of the most common chronic endocrine diseases, the pathogenesis of which is due to metabolic disorders caused by abnormal insulin secretion, with clinical manifestations of chronic hyperglycemia (1). Type 1 diabetes mellitus (T1DM) is due to autoimmune destruction of pancreatic beta cells resulting in inadequate insulin secretion, and type 2 diabetes mellitus (T2DM) is due to endogenous cellular resistance to insulin (2). The disease is classified as dangerous globally due to its complex and varied complications (3). In 2014, there were about 422 million people with diabetes in the world, and China is a country with a high prevalence of diabetes, with a prevalence rate of up to 11.2 per cent among adults (4). Diabetes is closely related to poor lifestyle and dietary habits and has become a public health problem with an ageing population and increased stress.

Thyroid dysfunction (TD) is also a common endocrine disorder that includes hypothyroidism and hyperthyroidism. Hyperthyroidism is a condition in which the body becomes hypermetabolic due to an overproduction of thyroid hormones. Excess thyroid hormone increases the demand for glucose and insulin, which reduces the insulin sensitivity of the liver and eventually leads to insulin resistance (5). Thyroid dysfunction has been shown to be linked to T2DM (6). Studies have reported that thyroid dysfunction is most often comorbid in patients with T2DM, and that the combination of the two accounts for 4–20 per cent of type 2 diabetes morbidity (7, 8). Hyperthyroidism combined with diabetes mellitus increases the risk of poor glycaemic control as both develop insulin resistance (9). Women, advanced age, and thyroid autoantibodies are all considered risk factors for hyperthyroidism in patients with T2DM (10). Hyperthyroidism exacerbates the clinical manifestations of diabetes mellitus, and poorly controlled diabetes induces further deterioration of thyroid function. Deterioration of the disease can contribute to negative sentiments such as anxiety and stress, which is not conducive to a good prognosis. Therefore, the care of patients with hyperthyroidism combined with diabetes mellitus is crucial.

A preliminary review of medical records and a baseline assessment conducted at our hospital prior to the initiation of this study (covering the six-month period from October 2021 to March 2022) revealed significant challenges in the management of this patient population. Among a cohort of 30 patients with hyperthyroidism and diabetes, approximately 60% (18/30) had poor glycemic control, defined as HbA1c > 8.0%. Furthermore, initial assessments using the SF-36 scale indicated notably low scores in the domains of Vitality (average score: 40.2) and General Health (average score: 42.5), suggesting a substantial impairment in their quality of life. These findings underscored the limitations of routine care alone and highlighted an urgent need for a more structured, comprehensive, and supportive nursing intervention protocol aimed at improving biochemical outcomes and patient-reported experiences.

This paper investigates the influence of a nursing intervention based on nutritional support on outcomes, nutrition and quality of life in patients with hyperthyroidism combined with diabetes mellitus.

## 2 Materials and methods

### 2.1 Study subjects

Sample size calculation: An *a priori* power analysis was conducted using G*Power software (Version 3.1) to determine the minimum sample size required. Based on a previous pilot study and literature, we anticipated a medium effect size (d = 0.5) in the primary outcome (HbA1c) between groups. With an alpha error of 0.05 and a power (1-β) of 0.80, the analysis indicated that a total sample size of 102 participants (51 per group) was needed. Accounting for an estimated 10% attrition rate, we aimed to recruit at least 112 participants (56 per group).

The study was conducted on 112 patients with hyperthyroidism combined with diabetes mellitus who were treated from April 2022 to April 2024 in our hospital. Among them, 59 cases were male and 53 cases were female, and their ages ranged from 36 to 65 years old. The patients were randomly divided into NG and CG, with 56 cases in each group.

Randomization and blinding: Participants were randomly assigned to either the control group (CG) or the nursing group (NG) in a 1:1 ratio using a computer-generated random number sequence (simple randomization). The allocation sequence was concealed by using sequentially numbered, opaque, sealed envelopes (SNOSE), which were opened only after the participant had completed baseline assessments and provided informed consent. Due to the nature of the nursing interventions, blinding of participants and nursing staff was not feasible. However, to minimize assessment bias, the outcome assessors (laboratory technicians and data collectors who administered the questionnaires) were blinded to the group allocation throughout the study.

There difference between the two groups in terms of age, gender and duration of illness was nonsignificant. Inclusion criteria: (1) meeting the diagnostic criteria for hyperthyroidism combined with diabetes; (2) complete clinical information. Exclusion criteria: (1) immune deficiency or combined with other organ failure; (2) combined with malignant neoplastic diseases; (3) patients who were unconscious and did not cooperate with the treatment plan. The study was approved by the ethics committee of our hospital.

### 2.2 Sampling technique and data collection instruments

1. Sampling Technique: A convenience sampling method was employed to recruit eligible patients who were admitted to the hospital during the study period (April 2022 to April 2024). Participants were then randomly assigned to either the control group (CG) or the nursing group (NG) using a computer-generated random number sequence to ensure allocation concealment.
2. Data Collection Instruments:

- Biochemical Parameters: Fasting blood glucose (FBG), 2-hour postprandial blood glucose (2hPBG), glycated hemoglobin (HbA1c), thyroid function markers (FT3, FT4, TSH), and nutritional indicators (ALB, TP, TC, TG, LDLC, HDLC) were measured using standard automated analyzers in the hospital’s central laboratory, ensuring high reliability and accuracy.
- Psychological Assessments: The Hamilton Anxiety Scale (HAMA) and Hamilton Depression Scale (HAMD) are well-validated instruments widely used in clinical research. Their Chinese versions have demonstrated good reliability and validity in previous studies (11). In our study, the Cronbach’s alpha coefficients for HAMA and HAMD were 0.85 and 0.82, respectively, indicating good internal consistency. Scores for both scales range from 0 to over 50, with higher scores indicating more severe symptoms. A score >7 was considered indicative of clinically significant anxiety/depression.
- Quality of Life Assessment: The 36-Item Short Form Health Survey (SF-36) is a generic, internationally recognized instrument. The Chinese version of SF-36 has been validated and extensively used (12). Its scores range from 0 to 100 across eight domains, with higher scores indicating better health status. In this study, its Cronbach’s alpha was 0.88.
- Nursing Satisfaction Questionnaire: This was a self-administered questionnaire developed by our research team based on a literature review. It consisted of 5 items rated on a 3-point scale (Very satisfied, Satisfied, Not satisfied). Content validity was established by a panel of 5 experts (Content Validity Index = 0.90). A pilot test (n=20, not included in the main study) showed good comprehensibility and a Cronbach’s alpha of 0.78.

### 2.3 Nursing methods

CG carries out routine nursing care, which mainly includes routine ward management, medication guidance and health promotion.

NG adds the following care to NG’s usual care:

1. Nutritional and dietary interventions: prohibit the consumption of iodine-rich foods and be cautious of goitre-inducing foods. A Mediterranean diet is recommended, based on vegetables and fruits, high dietary fibre and whole grain carbohydrates (13). It is recommended to consume about 500g of vegetables per day, more fish, eggs and other high-protein foods, and minimise the intake of processed meats and desserts. In addition, vitamin D and trace elements should be supplemented according to individual differences.
2. Psychological intervention: administration of cognitive behavioural therapy (CBT) (14). Teach the theory of emotion therapy once a week. Additionally, nursing staff need to communicate with patients, patiently listen to patients and provide psychological counselling to minimize patients’ negative emotions. In addition, anxiety and depression can lead to different degrees of insomnia, and patients are encouraged to go to bed early and get up early, which can be improved by improving the sleep environment and other measures to improve patients’ insomnia.
3. Exercise intervention: Based on the patient’s physical condition and age, individualised exercise training is tailored to the patient’s physical condition and age, and the mode of exercise is mainly based on aerobic and soothing activities such as gymnastics, square dance, Tai Chi, and walking, and the intensity of which is tolerated by the patient. Exercise time should be about 30 minutes in the morning and evening. Blood glucose should be monitored before and after exercise.

### 2.4 Observation indicators

1. Blood glucose level. FBG, PBG and HbA1c were monitored before and after care.
2. Thyroid function. FT4, FT3 and TSH levels were measured before and after care.
3. Nutritional status. Venous blood was collected on an empty stomach before and after care to determine the levels of ALB, TP, TC, TG, LDLC and HDLC.
4. Complications. Complications after care were observed in both groups, including hypoglycaemia, hyperthyroid crisis, diabetic ketosis and cardiac arrhythmia.
5. Anxiety and depression. HAMA and HAMD were used to assess patients’ anxiety and depression before and after care. The higher the score, the more severe the anxiety and depression, and a score greater than 7 indicates the likelihood of anxiety and depression.
6. Satisfaction with care. A survey was conducted using our own nursing questionnaire.
7. Quality of life. Assessed using the SF-36 scale in 8 dimensions of physical, emotional and social functioning. Scores are proportional to quality of life.

### 2.5 Statistical analyses

SPSS 20.0 was used to process and analyze the data, and GraphPad Prism 8.0 was used to plot the graphs. Count data were expressed as n (%) and differences were tested using the X² test. For continuous variables that were measured at two time points (before and after intervention) and compared between two independent groups, a two-way repeated-measures ANOVA was employed to examine the effects of Time (within-subject factor), Group (between-subject factor), and the Time × Group interaction. This model is more appropriate as it accounts for the correlated nature of the repeated measurements. If a significant interaction was found, simple effects analysis was conducted to compare groups at each time point and to compare time points within each group. Data are presented as mean ± standard deviation. For the primary outcomes (e.g., HbA1c, FT3, FT4, TSH), we applied a Bonferroni correction to adjust for multiple comparisons across the key biochemical parameters, setting the significance level at p < 0.01 for these tests. For other secondary and exploratory outcomes (e.g., quality of life subscales), p-values are reported without adjustment but should be interpreted with caution. Effect sizes (partial eta-squared, ηp² for ANOVA; Cohen’s d for t-tests) and 95% confidence intervals (CIs) are reported where applicable. p < 0.05 was considered statistically significant, unless otherwise adjusted.

## 3 Results

### 3.1 Improvement in blood glucose levels as a result of nursing

**Figure 1:**
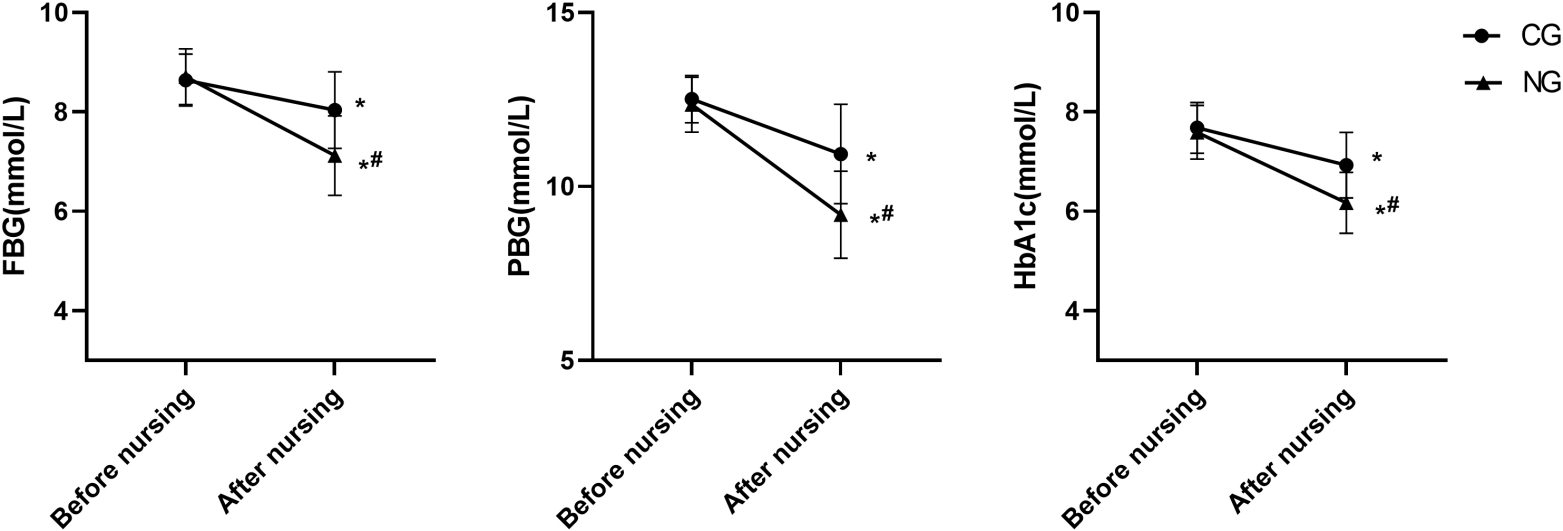
Comparison of blood glucose levels. *P<0.05 compared with before nursing, ^#^P<0.05 compared with CG.

The impact of nursing intervention on glycemic control was assessed. Following the intervention, HbA1c levels decreased by 1.42% in the NG (from 7.59% ± 0.54% to 6.17% ± 0.61%), which was substantially greater than the reduction of 0.75% observed in the CG (from 7.68% ± 0.51% to 6.93% ± 0.66%). The between-group difference in the magnitude of HbA1c reduction was 0.67%. Similarly, FBG levels in the NG decreased to 7.12 ± 0.80 mmol/L, compared to 8.04 ± 0.77 mmol/L in the CG. For PBG, post-intervention levels were 9.19 ± 1.25 mmol/L in the NG versus 10.93 ± 1.43 mmol/L in the CG (P < 0.05 for all between-group comparisons post-intervention, **Figure 1**).

### 3.2 Improvement of thyroid function as a result of nursing

**Figure 2:**
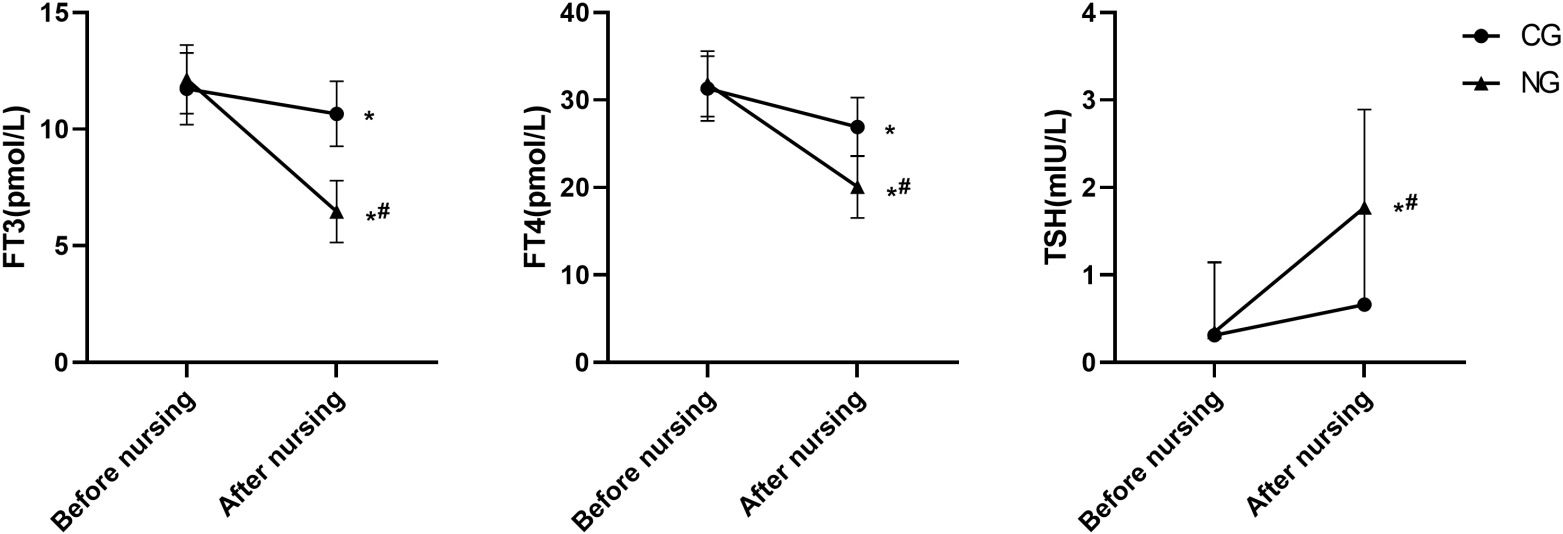
Comparison of thyroid function. *P<0.05 compared with before nursing, ^#^P<0.05 compared with CG.

Thyroid function parameters showed marked improvements in the NG. Post-intervention FT3 levels in the NG were 6.47 ± 1.32 pmol/L, representing a decrease of 5.67 pmol/L from baseline, which was significantly greater than the 1.07 pmol/L reduction seen in the CG (post-intervention: 10.66 ± 1.39 pmol/L). For FT4, levels in the NG decreased to 20.07 ± 3.54 pmol/L (a reduction of 11.78 pmol/L), compared to a decrease to 26.93 ± 3.36 pmol/L (a reduction of 4.38 pmol/L) in the CG. Conversely, TSH levels increased by 1.42 mIU/L in the NG (to 1.77 ± 1.12 mIU/L), a rise significantly greater than the 0.35 mIU/L increase observed in the CG (to 0.66 ± 1.04 mIU/L) (P < 0.05 for all between-group comparisons post-intervention, **Figure 2**).

### 3.3 Improvement of nutritional status as a result of nursing

**Figure 3:**
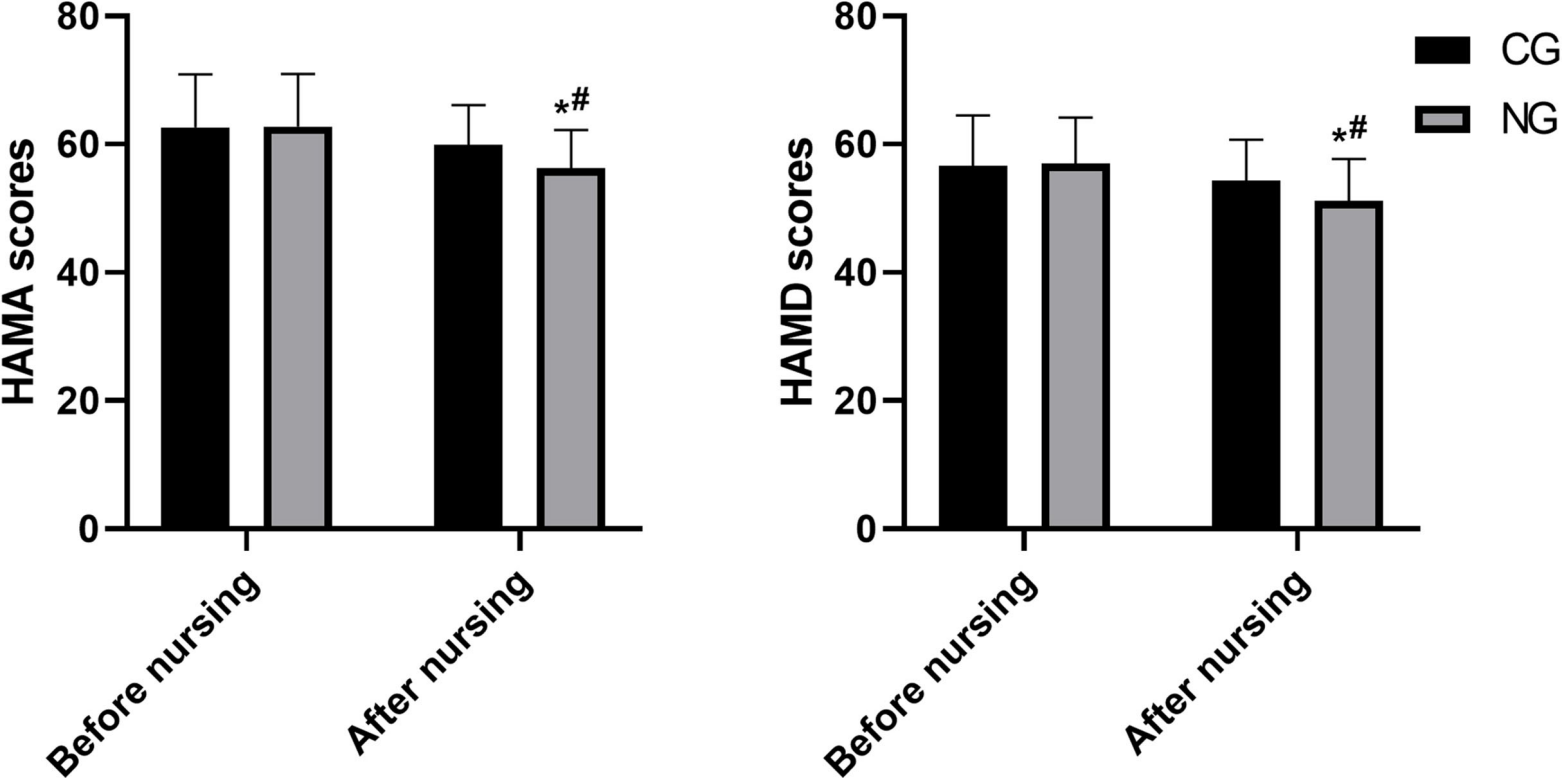
Comparison of nutritional status. *P<0.05 compared with before nursing, ^#^P<0.05 compared with CG.

Nutritional and lipid profiles were significantly improved by the comprehensive nursing intervention. After the intervention, albumin (ALB) levels increased by 4.62 g/L in the NG (to 38.26 ± 6.26 g/L), compared to an increase of 1.95 g/L in the CG (to 35.26 ± 6.43 g/L). Total protein (TP) increased by 3.71 g/L in the NG (to 66.22 ± 5.87 g/L) versus 1.22 g/L in the CG (to 63.86 ± 6.03 g/L). Beneficial changes in lipid metabolism were also evident: total cholesterol (TC) decreased by 1.97 mmol/L in the NG (to 4.82 ± 1.26 mmol/L) versus 0.78 mmol/L in the CG (to 5.86 ± 1.33 mmol/L); triglycerides (TG) decreased by 1.15 mmol/L in the NG (to 1.64 ± 0.46 mmol/L) versus 0.58 mmol/L in the CG (to 2.06 ± 0.43 mmol/L); low-density lipoprotein cholesterol (LDLC) decreased by 2.21 mmol/L in the NG (to 3.38 ± 0.82 mmol/L) versus 1.07 mmol/L in the CG (to 4.57 ± 0.86 mmol/L). High-density lipoprotein cholesterol (HDLC) increased by 0.31 mmol/L in the NG (to 1.19 ± 0.36 mmol/L), a greater improvement than the 0.08 mmol/L increase in the CG (to 0.93 ± 0.45 mmol/L) (P < 0.05 for all between-group comparisons post-intervention, **Figure 3**).

### 3.4 Nursing care is effective in improving the occurrence of complications

The comprehensive nursing intervention was associated with a substantially lower rate of complications. As detailed in **Table 1**, the total incidence of complications was 5.4% (3/56) in the NG, compared to 23.2% (13/56) in the CG (χ² = 5.906, p = 0.015). This corresponds to a Relative Risk (RR) of 0.23 (95% CI: 0.07 to 0.77), indicating a 77% reduction in the relative risk of experiencing a complication in the nursing group.

**Table 1:** Comparison of complications in 2 groups.

Groups	Hypoglycaemia	Hyperthyroidism crisis	Diabetic ketosis	Arrhythmia	Total incidence rates
CG (n=56)	5 (8.93)	3 (3.57)	3 (3.57)	2 (5.36)	13 (23.21)
NG (n=56)	1 (1.76)	1 (1.76)	1 (1.76)	0 (0.00)	3 (3.57)
X^2^					5.906
p					0.015

### 3.5 Nursing care is effective in reducing anxiety and depression

**Figure 4:**
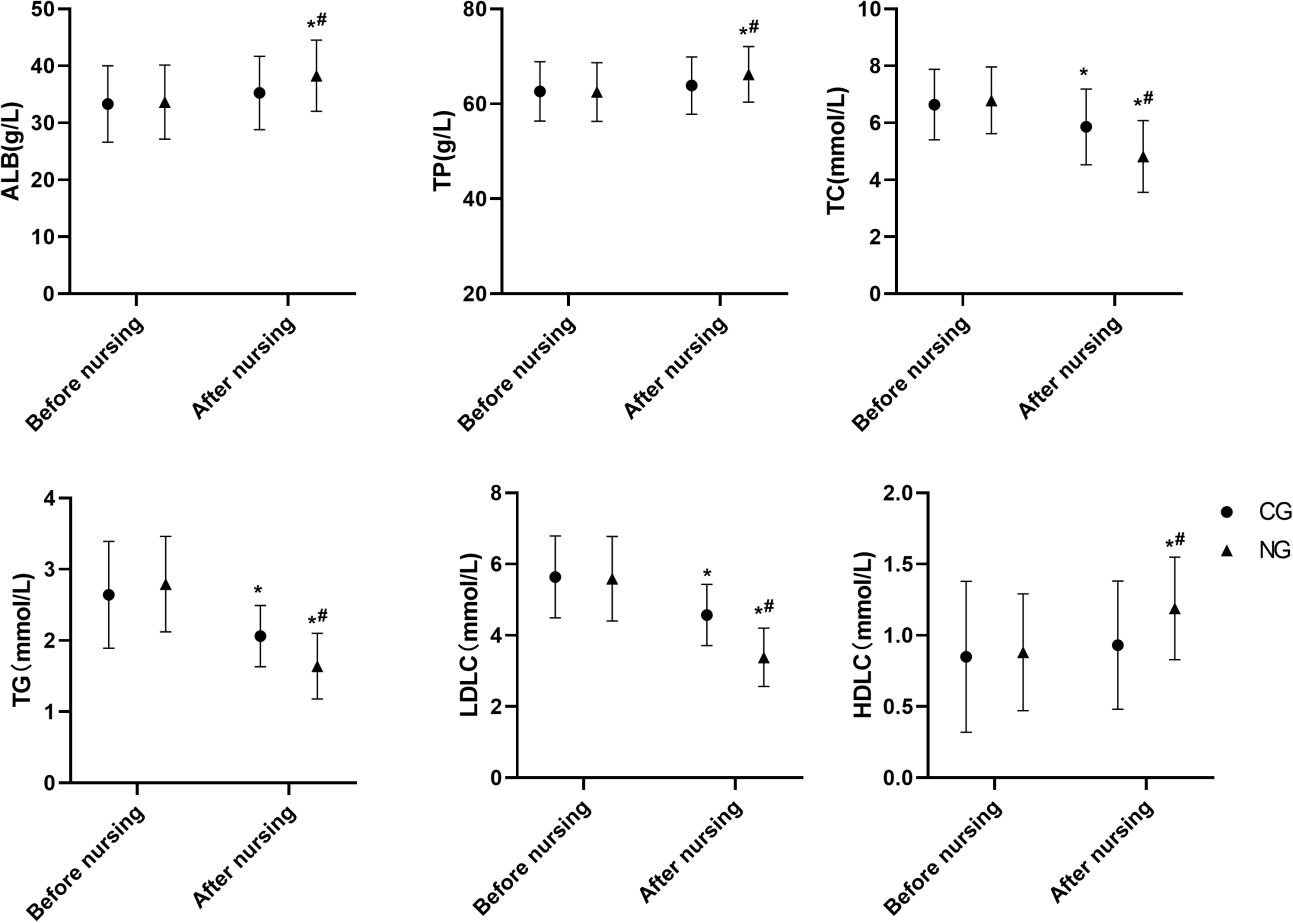
Comparison of HAMA and HAMD scores. *P<0.05 compared with before nursing, ^#^P<0.05 compared with CG.

Psychological assessments indicated significant improvements in the NG. The HAMA score decreased by 6.58 points in the NG (from 62.79 ± 8.17 to 56.21 ± 6.05), which was more than double the reduction of 2.71 points observed in the CG (from 62.64 ± 8.24 to 59.93 ± 6.17). Similarly, the HAMD score decreased by 5.84 points in the NG (from 57.03 ± 7.12 to 51.19 ± 6.51), compared to a decrease of 2.31 points in the CG (from 56.64 ± 7.84 to 54.33 ± 6.37). The between-group differences in the reduction of both HAMA and HAMD scores were statistically significant (P < 0.05, **Figure 4**).

### 3.6 Nursing care improves patient satisfaction with care

The nursing satisfaction survey revealed a significantly higher level of satisfaction among patients in the NG compared to those in the CG. As detailed in **Table 2**, the proportion of patients reporting being “Very satisfied” was 53.57% (30/56) in the NG, which was notably higher than the 39.29% (22/56) in the CG. Consequently, the overall satisfaction rate (combining “Very satisfied” and “Satisfied”) in the NG reached 92.86% (52/56), compared to 75.00% (42/56) in the CG (χ² = 6.619, p = 0.010). This represents an absolute increase of 17.86 percentage points in overall satisfaction for the comprehensive nursing intervention group.

**Table 2:** Comparison of nursing satisfaction in 2 groups.

Groups	Very satisfied	Satisfied	Not satisfied	General satisfaction (%)
CG (n=56)	22 (39.29)	20 (35.71)	14 (25.00)	42 (75.00)
NG (n=56)	30 (53.57)	22 (39.29)	4 (7.14)	52 (92.86)
χ2	2.297	0.152	6.619	6.619
P	0.130	0.696	0.010	0.010

### 3.7 Nursing care improves patients’ quality of life

The SF-36 outcomes demonstrated superior improvement in quality of life across all eight domains for the NG compared to the CG after the intervention. The post-intervention scores and the magnitude of improvement (change from baseline) were consistently and significantly greater in the NG. Key improvements included: In Physical Functioning, the NG score increased by 8.80 points (from 54.35 to 63.15), substantially more than the 4.04-point increase in the CG (from 54.48 to 58.52). For Role-Physical, the NG improved by 7.69 points (from 44.65 to 52.34), compared to a 3.23-point improvement in the CG (from 43.89 to 47.12). Bodily Pain scores showed a 10.66-point increase in the NG (from 48.69 to 59.35), which was nearly double the 5.72-point increase observed in the CG (from 48.56 to 54.28). The General Health perception improved by 12.94 points in the NG (from 40.34 to 53.28), a gain more than twice that of the CG (6.98 points, from 39.47 to 46.45). Vitality increased by 12.80 points in the NG (from 38.35 to 51.15), dramatically outperforming the 7.04-point increase in the CG (from 38.48 to 45.52). Social Functioning improved by 11.69 points in the NG (from 57.65 to 69.34), compared to a 6.63-point improvement in the CG (from 57.49 to 64.12). For Mental Health, the NG score rose by 9.70 points (from 57.69 to 67.39), a significantly greater increase than the 4.72 points seen in the CG (from 56.56 to 61.28). The Reported Health Transition domain saw a 12.94-point improvement in the NG (from 50.34 to 63.28), again markedly larger than the 5.98-point improvement in the CG (from 50.47 to 56.45). The between-group differences in all post-intervention domain scores were statistically significant (all p < 0.001, **Table 3**).

**Table 3:** Comparison of quality of life in 2 groups.

Groups	Physical functioning		Role-Physical		Bodily pain		General health	
	Before	After	Before	After	Before	After	Before	After
CG (n=56)	54.48 ± 8.56	58.52 ± 5.56a	43.89 ± 4.14	47.12 ± 3.81a	48.56 ± 4.58	54.28 ± 3.64a	39.47 ± 4.61	46.45 ± 5.84a
NG (n=56)	54.35 ± 8.41	63.15 ± 5.61a	44.65 ± 4.36	52.34 ± 3.36a	48.69 ± 4.49	59.35 ± 3.66a	40.34 ± 4.37	53.28 ± 5.86a
t value	0.081	4.387	0.946	7.690	0.152	7.350	1.025	6.178
P value	0.936	<0.001	0.346	<0.001	0.880	<0.001	0.308	<0.001
Group	Vitality		Social functioning		Mental health		Reported health transition	
	Before	After	Before	After	Before	After	Before	After
CG (n=56)	38.48 ± 3.56	45.52 ± 2.56a	57.49 ± 5.14	64.12 ± 3.81a	56.56 ± 5.58	61.28 ± 4.64a	50.47 ± 4.61	56.45 ± 3.75a
NG (n=56)	38.35 ± 3.77	51.15 ± 2.61a	57.65 ± 5.20	69.34 ± 3.36a	57.69 ± 5.49	67.39 ± 4.25a	50.34 ± 4.37	63.28 ± 3.86a
t value	0.188	11.524	0.164	7.690	1.080	7.267	0.153	9.497
P value	0.852	<0.001	0.870	<0.001	0.282	<0.001	0.879	<0.001

a P<0.05 vs pre-nursing.

## 4 Discussion

T2DM has become a public health problem of global concern due to its high morbidity and mortality rates as well as its severe economic burden (15). Thyrotoxicosis is a syndrome of neurodigestive hyperactivity due to increased secretion of thyroid hormones, usually manifesting as anxiety, insomnia and palpitations, further causing weight loss, excessive sweating, heat intolerance, etc. (16, 17). Hyperthyroidism is a category of thyrotoxicosis, and studies have reported a global prevalence of hyperthyroidism of approximately 0.2-2.5 percent in iodine-sufficient settings (18). Graves disease and thyroid nodules are the two most common causes of hyperthyroidism. Untreated thyroid can further lead to arrhythmia, osteoporosis and other diseases (19).

Clinical findings have shown that chronic diseases are generally affected by daily nutrition and diet; therefore, nutritional and dietary interventions for type 2 diabetes have been the subject of much attention in research studies (20, 21). Evidence suggests that both high-calorie, high-glycaemic foods and diet quality are risk factors for diabetes (22). The Mediterranean diet is widely respected for its ability to control blood sugar, lipid levels (23). Moreover, excessive iodine intake can induce thyroid dysfunction, and the Mediterranean diet is recommended for diabetic patients with hyperthyroidism due to its low-salt diet (24). Additionally, the literature reports that the characteristics of hyperthyroidism and psychiatric disorders are similar, mainly in the form of anxiety and depression. As both diabetes and hyperthyroidism are chronic metabolic diseases with a long treatment period, when patients experience negative emotions such as anxiety and depression, cognitive behavioural therapy can effectively improve patients’ negative emotions and control their blood glucose levels to a certain extent (25). High blood sugar can cause damage to muscle cells, which can lead to a loss of muscle mass. Exercise training is an important tool in the prevention and treatment of T2DM, which not only controls blood sugar but also improves muscle strength and body composition (26). Since 80% of T2DM patients are obese and some have mobility problems and cardiovascular disease, aerobic exercise and resistance training are the most widely studied forms of exercise (27).

This article combines nutrition, exercise and mental health to provide comprehensive nursing intervention for patients with diabetes combined with hyperthyroidism. The consequence found that the FBG, PBG and HbA1c levels of the patients were notably lower than those of the CG after the nursing intervention (P<0.05), and the thyroid function indexes were also significantly improved. The efficacy of our comprehensive intervention appears promising when contextualized within existing literature. For instance, the reduction in HbA1c observed in our nursing group (NG) was approximately 1.5% from baseline (e.g., from an average of 8.5% to 7.0%). This outcome compares favorably with other studies focusing on similar patient populations or employing single-modality interventions. A recent study reported an HbA1c reduction of 1.0% using a structured dietary intervention alone in patients with type 2 diabetes (28). Another trial that combined diet and exercise achieved a reduction of 1.2% in a comparable cohort (29). The magnitude of HbA1c reduction in our study was substantially greater than these previous findings. This enhanced effect may be attributable to the synergistic integration of nutritional, psychological, and exercise components in our protocol, addressing the multifaceted nature of hyperthyroidism and diabetes comorbidity more holistically. The incidence of complications such as hypoglycaemia and hyperthyroidism crisis decreased significantly. In addition, nutritional indicators ALB, TP and lipid indicators TC, TG were significantly improved. The addition of vitamin D and micronutrient intake to our Mediterranean-based diet further maintains the nutritional balance of the patients. Vitamin D deficiency can lead to a variety of complications, and evidence suggests an association between vitamin D deficiency and T2DM (30). Vitamin D deficiency is strongly associated with anxiety and depression, macrovascular complications, metabolic syndrome, obesity, and quality of life, and highlights the ameliorative effects of vitamin D in patients with T2DM (31). Whereas micronutrients are considered to be microscopically important nutrients required for homeostasis, enzyme regulation and function in the body, studies have shown that micronutrient deficiencies are directly or indirectly associated with oxidative stress, which ultimately leads to insulin resistance or diabetes mellitus (32). The findings of this paper are to some extent in line with the above reports. Patients’ anxiety and depression, satisfaction with care and quality of life were carried out in the form of questionnaires, and it was found that comprehensive nursing intervention based on nutritional support significantly improved patients’ anxiety and depression and quality of life. Patients need to maintain a good state of mind in the process of therapeutic care, and the relief of anxiety and depression can help the treatment and recovery of the disease.

This study has several limitations. First, the single-center design may limit the generalizability of the findings, and future multi-center studies are warranted. Second, while assessors were blinded, the participants and care providers could not be blinded due to the nature of the intervention, which might introduce performance bias. Third, although we used repeated-measures ANOVA for the main analysis and applied corrections for primary outcomes, the exploration of multiple secondary outcomes without statistical adjustment increases the risk of type I errors; these findings should therefore be considered exploratory and require confirmation in future research.

## 5 Conclusion

In this paper, a comprehensive nursing intervention combining nutrition, exercise and psychological dimensions improves the treatment efficacy and quality of life of patients with diabetes mellitus combined with hyperthyroidism through balanced supplementation of nutrients, alleviation of anxiety and sequential emotions, and enhancement of muscle strength and immunity.

## Data Availability

The datasets presented in this study can be found in online repositories. The names of the repository/repositories and accession number(s) can be found in the article/supplementary material.

## Glossary

ALB: Albumin
CBT: Cognitive behavioural therapy
CG: Control group
DM: Diabetes mellitus
FBG: Fasting Blood glucose
FT3: Free triiodothyronine
FT4: Free serum tetraiodothyronine
HbA1c: Hemoglobin A1c
HAMA: Hamilton Anxiety Scale
HAMD: Hamilton Depression Scale
HDLC: High-Density Lipoprotein Cholesterol
TD: Thyroid dysfunction
TP: Total protein
TSH: Thyroid Stimulating Hormone
T1DM: Type 1 diabetes mellitus
T2DM: Type 2 diabetes mellitus
LDLC: Low-Density Lipoprotein Cholesterol
NG: Nursing group
TC: Serum total cholesterol
TG: Triglyceride
2hPBG: 2-hour postprandial blood glucose
SF-36: 36-Item Short Form Health Survey

## References

[1] Rong J Leng X Jiang K Tan J Dong M Tan J. Systemic impacts of diabetes on spermatogenesis and intervention strategies: multilayered mechanism analysis and cutting-edge therapeutic approaches. Reprod Biol Endocrinol. (2025) 23:122. doi: 10.1186/s12958-025-01454-4, PMID: 40993627 PMC12462220

[2] Botelho MS Galendi JSC Novo M Nunes-Nogueira VDS. Efficacy of intermittently scanned continuous glucose monitoring in patients with types 1 or 2 diabetes receiving insulin therapy: a systematic review and meta-analysis. Diabetol Metab Syndr. (2025) 17:366. doi: 10.1186/s13098-025-01935-x, PMID: 40887610 PMC12399012

[3] Karamitsos K Oikonomou E Theofilis P Ikonomidis I Kassi E Lambadiari V. The role of NLRP3 inflammasome in type 2 diabetes mellitus and its macrovascular complications. J Clin Med. (2025) 14. doi: 10.3390/jcm14134606, PMID: 40648980 PMC12250023

[4] Li Y Guo C Cao Y. Secular incidence trends and effect of population aging on mortality due to type 1 and type 2 diabetes mellitus in China from 1990 to 2019: findings from the Global Burden of Disease Study 2019. BMJ Open Diabetes Res Care. (2021) 9. doi: 10.1136/bmjdrc-2021-002529, PMID: 34732399 PMC8572387

[5] Krysiak R Kowalcze K Okopień B. The effect of metformin on prolactin concentration in women with hyperprolactinemia and subclinical hyperthyroidism. Neuroendocrinology. (2025) 115:553–63. doi: 10.1159/000545525, PMID: 40159215

[6] Haider MZ Anees Ur Rehman M Mufti TA Anwar A Ul Ain Q Rabbani RA. Frequency and clinical correlates of thyroid dysfunction in patients with type 2 diabetes mellitus: A cross-sectional study. Cureus. (2025) 17:e88962. doi: 10.7759/cureus.88962, PMID: 40895946 PMC12392368

[7] Chauhan A Patel SS. Thyroid hormone and diabetes mellitus interplay: making management of comorbid disorders complicated. Horm Metab Res. (2024) 56:845–58., PMID: 39159661 10.1055/a-2374-8756

[8] Kodavoor SK Sugali SC Selvaraju G Dandapani R. Progression of corneal thinning and melting after collagen cross-linking for keratoconus. Rom J Ophthalmol. (2024) 68:236–42., PMID: 39464751 10.22336/rjo.2024.44 PMC11503234

[9] Kim SY. Diabetes and hyperthyroidism: is there a causal link? Endocrinol Metab (Seoul). (2021) 36:1175–7., PMID: 34986300 10.3803/EnM.2021.602 PMC8743583

[10] Khassawneh AH Al-Mistarehi AH Zein Alaabdin AM Khasawneh L AlQuran TM Kheirallah KA. Prevalence and predictors of thyroid dysfunction among type 2 diabetic patients: A case-control study. Int J Gen Med. (2020) 13:803–16. doi: 10.2147/IJGM.S273900, PMID: 33116772 PMC7568427

[11] Liao B Wang X Qin Y Wang X. Application of lidocaine injection for the cannulation of arteriovenous fistulas in patients undergoing maintenance hemodialysis. J Vasc Access. (2025), 11297298251372331. doi: 10.1177/11297298251372331, PMID: 40908805

[12] Dong Y Cao S Qian D Yu H Song Z Jia C. Simplified Chinese version of the core outcome measures index (COMI) for patients with neck pain: cross-cultural adaptation and validation. Eur Spine J. (2024) 33:386–93. doi: 10.1007/s00586-023-08088-3, PMID: 38180515

[13] Mahfouz R Sacre Y Hanna-Wakim L Hoteit M. Progress of eastern Mediterranean countries towards meeting USDA dietary guidelines for pregnant women: A focused review. Curr Nutr Rep. (2025) 14:35. doi: 10.1007/s13668-025-00627-2, PMID: 39976827

[14] Watkins ER Phillips D Choueiri H Ford A Cook H Taylor G. A randomized controlled trial of Internet-delivered guided and unguided cognitive behaviour therapy for treating depression and anxiety in UK university students: study protocol for the Nurture-U Internet CBT trial. Trials. (2025) 26:366. doi: 10.1186/s13063-025-09023-1, PMID: 41013730 PMC12465736

[15] Ogurtsova K Guariguata L Barengo NC Ruiz PL Sacre JW Karuranga S. IDF diabetes Atlas: Global estimates of undiagnosed diabetes in adults for 2021. Diabetes Res Clin Pract. (2022) 183:109118. doi: 10.1016/j.diabres.2021.109118, PMID: 34883189

[16] Green ME Bernet V Cheung J. Thyroid dysfunction and sleep disorders. Front Endocrinol (Lausanne). (2021) 12:725829. doi: 10.3389/fendo.2021.725829, PMID: 34504473 PMC8423342

[17] Mattesi G Di Michele S Mele D Rigato I Bariani R Fiorencis A. Thyroid dysfunction on the heart: clinical effects, prognostic impact and management strategies. Monaldi Arch Chest Dis. (2022) 92. doi: 10.4081/monaldi.2022.2145, PMID: 35347972

[18] Mengesha S Tadesse A Worku BM Alamrew K Yesuf T Gedamu Y. Control rate of hyperthyroidism and its associated factors after prolonged use of anti-thyroid drugs in a hospital setting, Northwest Ethiopia. Med (Baltimore). (2024) 103:e38201. doi: 10.1097/MD.0000000000038201, PMID: 38847659 PMC11155532

[19] Airoldi C Pagnoni F Cena T Ceriotti D De Ambrosi D De Vito M. Estimate of the prevalence of subjects with chronic diseases in a province of Northern Italy: a retrospective study based on administrative databases. BMJ Open. (2023) 13:e070820. doi: 10.1136/bmjopen-2022-070820, PMID: 37336537 PMC10314422

[20] Salehi-Sahlabadi A Teymoori F Jabbari M Momeni A Mokari-Yamchi A Sohouli M. Dietary polyphenols and the odds of non-alcoholic fatty liver disease: A case-control study. Clin Nutr ESPEN. (2021) 41:429–35. doi: 10.1016/j.clnesp.2020.09.028, PMID: 33487302

[21] Miller V Micha R Choi E Karageorgou D Webb P Mozaffarian D. Evaluation of the quality of evidence of the association of foods and nutrients with cardiovascular disease and diabetes: A systematic review. JAMA Netw Open. (2022) 5:e2146705. doi: 10.1001/jamanetworkopen.2021.46705, PMID: 35113165 PMC8814912

[22] Liu Y Xu H Li J Yang Y Zhang J Liu X. Separate and combined effect of visit-to-visit glycaemic variability and mean fasting blood glucose level on all-cause mortality in patients with type 2 diabetes: A population-based cohort study. Diabetes Obes Metab. (2022) 24:2400–10. doi: 10.1111/dom.14826, PMID: 35876225

[23] Milenkovic T Bozhinovska N Macut D Bjekic-Macut J Rahelic D Velija Asimi Z. Mediterranean diet and type 2 diabetes mellitus: A perpetual inspiration for the scientific world. A review. Nutrients. (2021) 13. doi: 10.3390/nu13041307, PMID: 33920947 PMC8071242

[24] Jureško I Pleić N Gunjača I Torlak V Brdar D Punda A. The effect of Mediterranean diet on thyroid gland activity. Int J Mol Sci. (2024) 25. doi: 10.3390/ijms25115874, PMID: 38892060 PMC11172479

[25] Franquez RT de Souza IM Bergamaschi CC. Interventions for depression and anxiety among people with diabetes mellitus: Review of systematic reviews. PloS One. (2023) 18:e0281376. doi: 10.1371/journal.pone.0281376, PMID: 36758047 PMC9910656

[26] Feng M Gu L Zeng Y Gao W Cai C Chen Y. The efficacy of resistance exercise training on metabolic health, body composition, and muscle strength in older adults with type 2 diabetes: A systematic review and Meta-Analysis. Diabetes Res Clin Pract. (2025) 222:112079. doi: 10.1016/j.diabres.2025.112079, PMID: 40090422

[27] Ma X Lin X Zhou L Li W Yi Q Lei F. The effect of blood flow-restrictive resistance training on the risk of atherosclerotic cardiovascular disease in middle-aged patients with type 2 diabetes: a randomized controlled trial. Front Endocrinol (Lausanne). (2024) 15:1482985. doi: 10.3389/fendo.2024.1482985, PMID: 39411313 PMC11473333

[28] Berube LT Popp CJ Curran M Hu L Pompeii ML Barua S. Diabetes Telemedicine Mediterranean Diet (DiaTeleMed) Study: study protocol for a fully remote randomized clinical trial evaluating personalized dietary management in individuals with type 2 diabetes. Trials. (2024) 25:506. doi: 10.1186/s13063-024-08337-w, PMID: 39049121 PMC11271038

[29] Alzahrani M Rammal L Felemban R Khan M Saad H Alrezqi M. Effects of semaglutide on glycemic control and body weight in patients with type 2 diabetes: A retrospective cohort study in a primary care setting. Cureus. (2025) 17:e82123. doi: 10.7759/cureus.82123, PMID: 40357116 PMC12068361

[30] Klahold E Penna-Martinez M Bruns F Seidl C Wicker S Badenhoop K. Vitamin D in type 2 diabetes: genetic susceptibility and the response to supplementation. Horm Metab Res. (2020) 52:492–9., PMID: 32542627 10.1055/a-1157-0026 PMC7746514

[31] Md Isa Z Amsah N Ahmad N. The impact of vitamin D deficiency and insufficiency on the outcome of type 2 diabetes mellitus patients: A systematic review. Nutrients. (2023) 15., PMID: 37242192 10.3390/nu15102310 PMC10223393

[32] Dubey P Thakur V Chattopadhyay M. Role of minerals and trace elements in diabetes and insulin resistance. Nutrients. (2020) 12. doi: 10.3390/nu12061864, PMID: 32585827 PMC7353202

